# Pulsed Electric Field-Induced Ablation of Atherosclerotic Plaque-Forming Cells: A Preliminary In Vitro Investigation

**DOI:** 10.3390/jcdd13100515

**Published:** 2026-10-13

**Authors:** Yongle Jing, Zhixiao Xue, Xinwei Cui, Yuli Wu, Xuying Ye, Chengzhi Lu, Huijuan Yin, Manxi Liu, Xiaoqiang Sun, Ziyi Zhang

**Affiliations:** 1 Department of Cardiology, Tianjin First Central Hospital, No. 24, Fukang Road, Tianjin 300192, China; yonglejing@tmu.edu.cn (Y.J.); 5020201787@nankai.edu.cn (X.S.); 2 School of Biomedical Engineering and Technology, Tianjin Medical University, No. 22, Qixiangtai Road, Tianjin 300070, China; xuezhixiao@126.com (Z.X.); cuikaifa666@163.com (X.C.); 3 Department of Anesthesiology, Tianjin First Central Hospital, No. 24, Fukang Road, Tianjin 300192, China; wyl2023@nankai.edu.cn; 4 Institute of Biomedical Engineering, Chinese Academy of Medical Sciences & Peking Union Medical College, No. 236, Baidi Road, Tianjin 300192, China; yinhj@bme.pumc.edu.cn; 5 Clinical Medicine College of Tianjin Medical University, No. 22, Qixiangtai Road, Tianjin 300070, China; lmx060525@163.com; 6 Radiology of Beijing United Family Hospital of Integrative Medicine Co., Ltd., No. 46, West Headquarters Hutong, Beijing 100016, China; zhang_ziyi@163.com

**Keywords:** pulsed electric field, atherosclerosis, foam cells, macrophages, smooth muscle cells

## Abstract

Background: Pulsed electric field (PEF) has been investigated as a local ablation modality in various biomedical applications. Atherosclerotic plaques are composed of pathological cells, including macrophages, vascular smooth muscle cells (SMCs) and foam cells. The in vitro ablation effects of PEF on these plaque-forming cells remain incompletely characterized. Methods: Mouse RAW 264.7 macrophages, MOVAS SMCs, and foam cells derived from both lines were treated with PEF (250–1000 V/cm). Cell death was assessed by live–dead staining. Surface ultrastructural changes were examined by scanning electron microscopy (SEM). Membrane phosphatidylserine externalization was evaluated by Annexin V-FITC/propidium iodide (PI) staining. Expression changes in apoptosis-associated proteins (Bax, Bcl-2, Caspase-3, Cytochrome C) were measured by Western blot. Secretion of selected cytokines (IL-1β, TNF-α, IFN-γ, IL-10, TGF-β) was measured by enzyme-linked immunosorbent assay (ELISA) at 24 h. Results: PEF induced dose-dependent reductions in viability in all tested cell types. SEM revealed pronounced membrane ultrastructural changes, including disruption of the surface meshwork and pore-like features. The proportion of Annexin V-positive cells increased after PEF treatment. The expression levels of apoptosis-associated proteins and their statistical significance varied by cell type and time point. No statistically significant differences were detected in the selected cytokines at 24 h under the tested conditions. Conclusion: Under the tested in vitro conditions, PEF induced dose-dependent ablation effects on atherosclerotic plaque-forming cells, accompanied by membrane ultrastructural alterations and increased Annexin V-positive cell proportions. These findings provide preliminary in vitro evidence warranting further investigation in more physiologically relevant models.

## 1. Introduction

Atherosclerosis is a major causative factor of cardiovascular disease worldwide, and its core pathology is characterized by the formation of plaques from lipid deposition in the arterial lining. This leads to stenosis and even occlusion of blood vessels, which in turn lead to atherosclerotic cardiovascular diseases (ASCVDs), such as coronary heart disease, stroke and peripheral arterial diseases, which are serious threats to human health [1]. The treatment of atherosclerotic diseases relies heavily on traditional pharmaceutical antiplatelet aggregation, lipid-lowering and anti-inflammatory strategies, as well as revascularization treatment [2]. However, these approaches have limitations. Although medication can slow plaque progression, it has a limited effect on removing plaque that has already formed. Stent implantation, on the other hand, maintains vascular patency through mechanical support, but the stent-associated chronic inflammatory response and endothelial hyperplasia are important risk factors for recurrence. Therefore, exploring new treatments to reduce plaque load, minimize recurrence, and reduce the risk of complications has become a focus of research.

In recent years, pulsed electric field (PEF) has attracted increasing attention because of its unique therapeutic mechanism and relatively minor side effects. The basic principle of PEF ablation is to utilize high-voltage pulses for a short period of time, resulting in electroporation of cell membranes, which in turn induces cell death [3]. Studies have suggested that PEF ablation can ablate diseased tissues while largely sparing the surrounding normal tissues in certain settings [4,5]. The application of this technology in tumor treatment has achieved remarkable results [6]. In addition, PEF ablation has shown good results in the ablation treatment of atrial fibrillation [7]. In a recent study, Chang et al. demonstrated that PFA could safely and effectively kill septal cardiomyocytes and reduce septal mass without significant acute edema or severe arrhythmias in the ablated area [8]. While the ablation effect of PEF via electroporation is established in other fields, its application to atherosclerosis therapy hinges on a distinct premise: the need for a controlled ablation that minimizes plaque-destabilizing inflammation. Therefore, this preliminary in vitro investigation was designed to characterize the in vitro ablation effects of PEF on key atherosclerotic plaque-forming cells—macrophages, SMCs, and foam cells. Specifically, we examined (a) dose-dependent changes in cell viability, (b) surface ultrastructural alterations, (c) Annexin V-positive cell proportions, (d) expression changes in apoptosis-associated proteins, and (e) secretion of selected inflammatory cytokines. The goal was to provide foundational descriptive in vitro data on PEF-induced ablation phenomena without extrapolating to clinical therapeutic claims.

## 2. Materials and Methods

### 2.1. Cell Culture and Foam Cell Model Construction

The mouse monocytic leukemia cell line RAW 264.7 and the mouse vascular SMC line MOVAS (both purchased from Procell Life Science Co., Ltd., Wuhan, China) were inoculated at 5 × 10^4^ cells/mL in 10 cm dishes, cultured in complete Dulbecco’s modified eagle medium (DMEM) (containing 10% fetal bovine serum and 1% penicillin/streptomycin/amphotericin B) in an incubator at 37 °C and 5% CO_2_, and passaged on alternate days. In this study, macrophage-derived and SMC-derived foam cell models were established with the above 2 cell lines. RAW 264.7 cells and MOVAS cells were passaged and cultured, and after they entered the logarithmic growth phase, they were induced with oxidized low-density lipoprotein (ox-LDL) at 100 µg/mL for 24 h. Oil red O staining was used to visualize the intracellular lipid droplets. Foam cell modeling was considered successful if the cells contained a large number of lipid droplets and if the cytoplasmic volume was enlarged, features consistent with the morphological characteristics of foam cells.

### 2.2. Cellular Ablation Systems

**Figure 1:**
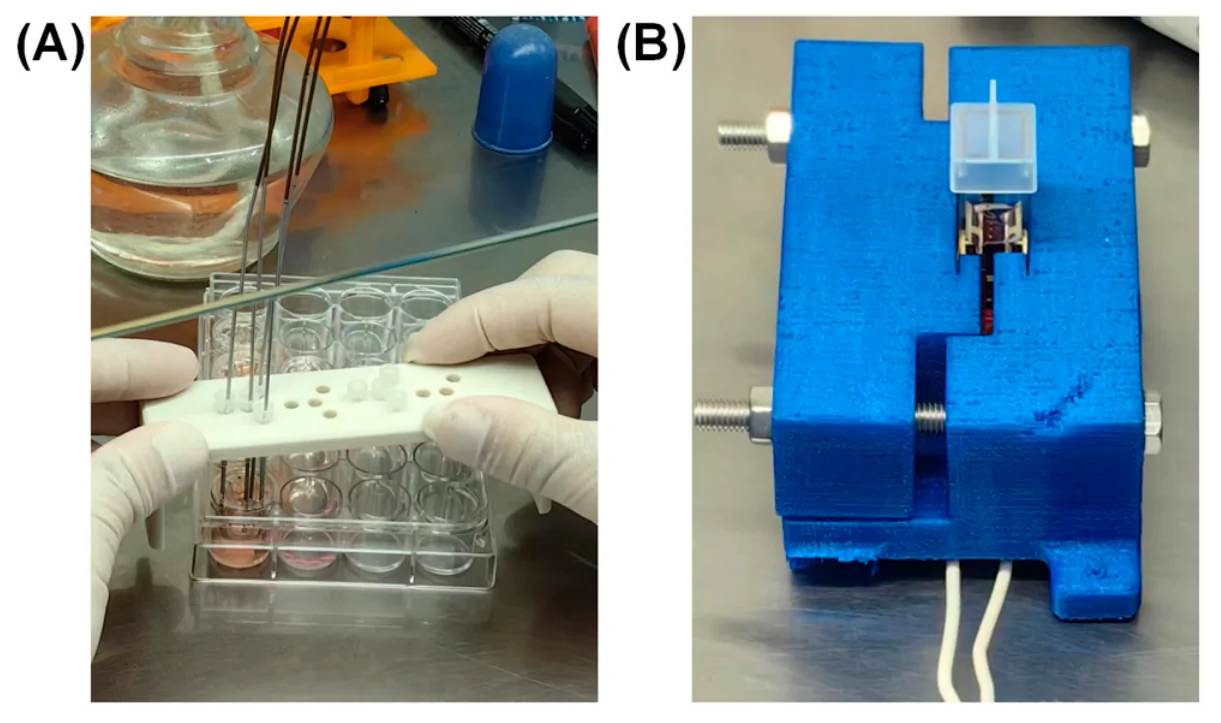
Schematic diagrams of the two PEF ablation systems used in this study. (**A**) Monolayer adherent cell ablation system. This system is designed to simulate the ablation of cells in a tissue-like, adherent state. (**B**) Single-cell suspension ablation system. This system allows for the efficient and uniform delivery of PEF to a high density of cells and was used for experiments requiring larger cell yields. PEF: pulsed electric field.

A monolayer adherent cell ablation system and a single-cell suspension ablation system previously constructed by our team were used to conduct targeted studies according to different experimental purposes and needs (Figure 1).

The monolayer adherent cell ablation system involves inoculating RAW 264.7 cells and MOVAS cells at a density of 5 × 10^5^ cells/mL onto the membranes of cell culture chambers placed in a 24-well plate. An appropriate amount of medium is added both inside and outside the chambers, and after 24 h of incubation, the cells are induced to develop into foam cells with 100 µg/mL ox-LDL for 24 h, during which they grow into dense monolayers of cell membranes. For the PEF intervention, four electrodes are used, including one positive electrode suspended vertically in the culture chambers. Three negative electrodes are inserted into the outside of the chambers through three holes around the culture chambers. A conical discharge is formed between the positive and negative electrodes. The top side of the cell layer is positive, and the bottom side of the cell is negative. Owing to the 5 μm holes in the membrane of the culture chambers, it is possible to ensure that the cells do not leak down and that the upper and lower poles of the cells are directly exposed to the electric fields during ablation. This method can better simulate the physiological and pathological states of adherent cells.

Owing to the limited volume of the cell chambers in the monolayer adherent cell ablation system, the number of cultured cells could not meet the demands of some cell experiments. Thus, we also used a single-cell suspension ablation system. This method involves growing RAW 264.7, MOVAS and foam cells in Petri dishes, and then transferring them at a density of 5 × 10^5^ cells/mL to electroshock cups (4 mm spacing). An electroshock cup has a volume of 1 mL and is equipped with 2 parallel conductive aluminum plates molded in seamless plastic to avoid leakage and ensure consistent pulse delivery to the sample. The electroshock cup is placed in the ablation tank, the positive and negative electrodes of the PEF ablator are connected and fixed, and then the PEF treatment is performed according to predetermined parameters. In this study, live–dead cell staining was performed using the monolayer adherent ablation system, while flow cytometry, SEM, Western blot and enzyme-linked immunosorbent assay (ELISA) were performed using the single-cell suspension ablation system.

### 2.3. Pulsed Electric Field Ablation Parameter Settings

The pulse generator is produced by Tianjin Eagle Terry Ankang Medical Technology Co. (Tianjin, China). The system delivers electric fields from 500 to 3000 V, with up to 100 pulses and a maximum pulse length of 100 milliseconds. The electric field parameters are the pulse mode, field strength, action time, pulse width, number of pulses and pulse frequency. To explore appropriate ablation protocols for atheromatous plaques, we implemented five different high-frequency current stimulation intensities with peak amplitudes of 250 V/cm, 350 V/cm, 500 V/cm, 800 V/cm, and 1000 V/cm. The field strengths (250–1000 V/cm) were calculated from the applied voltage and electrode distance. For the monolayer adherent cell ablation system, the electrode gap was 5 mm. For the suspension ablation system, the electrode gap was 4 mm. Current, conductivity, delivered energy, and temperature were not recorded in the present system. All the protocols were performed using biphasic square-wave pulses with a pulse frequency of 50,000 hertz (Hz). The pulse mode was set to short asymmetric pulses in both directions. The positive pulse width was 7 µs with a 5 µs pulse interval, and the negative pulse width was 3 µs with a 5 µs pulse interval. Each ablation consisted of a total of 10 pulse cycles, each consisting of 10 bidirectional pulses.

### 2.4. Cellular Experimental Methods

All cellular experiments were performed with three independent biological replicates (cells from separate cultures and treatments). Live–dead cell staining was used to assess cell viability at 24 h following PEF treatment. Cells in chamber slides were treated with PEF and incubated for 24 h. After discarding the medium, the chambers were placed into a 24-well plate. A mixture of 1 µM calcein AM and 1 µM PI was added, and cells were stained at room temperature for 30 min. After washing with PBS, live (green, calcein AM-positive) and dead (red, PI-positive) cells were observed and imaged under a light microscope.

Scanning electron microscopy (SEM) was performed to observe PEF-induced alterations in cellular surface morphology. Following PEF treatment, RAW 264.7 and MOVAS cells were incubated for 24 h. The cells were then pelleted, resuspended, and centrifuged to form a cell pellet. The supernatant was discarded, and the cells were fixed overnight with 2.5% glutaraldehyde at 4 °C. Cell surface morphology was then observed using a biological SEM (ZEISS Sigma 360) (Carl Zeiss AG, Oberkochen, Germany).

Annexin V-FITC/propidium iodide (PI) double staining was employed to quantify the proportions of Annexin V-positive cells. RAW 264.7, MOVAS and foam cells were treated with PEF under the conditions described above, transferred to 24-well culture dishes, cultured for 24 h, prepared into single-cell suspensions, incubated with Annexin V-FITC and PI immediately as per the instructions, added to the corresponding 1.5 mL EP tubes. The cell concentration was adjusted to approximately 5 × 10^5^ cells/mL in a final volume of 100 µL. Prior to analysis, each sample was filtered through a 40 µm (300-mesh) cell strainer. Flow cytometry was performed on a BD FACSVerse^TM^ system (BD Biosciences, San Jose, CA, USA). Data were acquired using standardized instrument settings, and a minimum of 35,000 events were recorded per sample. The gating strategy was applied as follows: cells were first gated based on forward scatter (FSC-A) and side scatter (SSC-A) to exclude debris and non-cellular events. A stringent polygon gate was then applied to select the main cell population. Compensation and quadrant thresholds were established using single-stained controls (Annexin V single-staining, PI single-staining, and unstained controls). Conventional singlet discrimination (FSC-H vs. FSC-A) was not performed because pulse geometry parameters were not available. This is a technical limitation. A strict FSC/SSC gating strategy was used to minimize the inclusion of cell aggregates. Subsequently, the proportion of Annexin V-positive cells was assessed on the Annexin V-FITC vs. PI dot plot. Quadrant analysis distinguished the following populations: Annexin V−/PI− (viable), Annexin V+/PI− (early apoptotic), Annexin V+/PI+ (mixed population that may include late apoptotic and secondary necrotic cells), and Annexin V−/PI+ (necrotic). All gates were applied consistently across all experimental groups. The acquired flow maps were processed with FlowJo V 10 software. Because Annexin V+/PI+ cells may include both late apoptotic and secondary necrotic cells, the data are reported descriptively as Annexin V-positive cell proportions without asserting a specific mode of cell death.

Western blot was utilized to evaluate the expression levels of apoptosis-associated proteins. RAW 264.7, MOVAS and foam cells were treated with PEF under the conditions described above and then cultured for 2 h and 24 h, after which the medium was removed, the cells were washed with PBS twice, RIPA lysis buffer was added, and the culture dish was shaken repeatedly so that the cells were in full contact with the lysis buffer. The cells were then scraped from the dish with a cell scraper into 1.5 mL EP tubes and allowed to stand for 5 min to ensure complete lysis, after which the samples were centrifuged at 12,000 rpm for 20 min. After centrifugation, the supernatant from the EP tube was aspirated into a new EP tube, the sampling buffer was added, the sample was heated at 100 °C for 10 min to denature the protein, and the protein samples were stored at −20 °C until use. After thawing, the samples were separated by SDS–PAGE and transferred to a membrane. After blocking, the membrane was incubated with primary antibodies (anti-Bax antibody, anti-Bcl-2 antibody, anti-Caspase-3 antibody, and anti-Cytochrome C antibody), after which the membrane was incubated with horseradish peroxidase (HRP)-labeled secondary antibody at room temperature on a shaker. After the membrane was washed, it was developed with enhanced chemiluminescence (ECL) and a chemiluminescence instrument was used to visualize the protein bands, which were analyzed using Image J 1.54 p software to obtain the grayscale values of the bands. Thus, the relative protein content was calculated. Because the antibodies recognize total Caspase-3 and total Cytochrome C, the data reflect total protein levels and do not distinguish cleaved Caspase-3 or cytosolic Cytochrome C.

ELISA was conducted to measure the secretion of inflammatory cytokines. The cell culture supernatant was collected 24 h after PEF ablation and centrifuged at 3000 rpm for 10 min to remove the cell sediment, after which the supernatant was collected. Ten wells of the standard were set up according to the instructions of the kit, and the samples were sequentially diluted and added. Blank wells and sample wells were set up separately; 40 µL of sample dilution was added to the sample airspace of the sample to be tested, and then 10 µL of the sample to be tested was added to the sample airspace of the enzyme-coated plate, and after sealing the plate it was incubated at 37 °C for 30 min. The seal was removed and the liquid was discarded; each well was filled with washing solution, shaken for 30 s, and then the liquid was discarded, which was repeated five times. After gently blotting with filter paper to remove all remaining liquid, 50 µL of enzyme labeling reagent was added to each well, except for the blank wells, and the samples were incubated and washed again. After the samples were washed, 50 µL of color developer A was added, 50 µL of color developer B was added, it was mixed gently, and the color was developed at 37 °C for 15 min in the dark. The termination solution was added to terminate the reaction; at this time, the blue color immediately turned yellow. The plate reader was calibrated to zero with a blank well, and the absorbance (OD value) of each well was measured at 450 nm. The concentrations of IL-1β, TNF-α, IFN-γ, IL-10 and TGF-β in the samples were calculated from the standard curve.

### 2.5. Experimental Controls, Reproducibility, and Variability

To ensure the robustness and reproducibility of our findings, the following measures were implemented throughout the study.

Standardization of procedures: All cell lines were maintained within a defined passage range (passages 5–15 for RAW 264.7 and MOVAS) to ensure phenotypic consistency. The PEF ablation procedure for all comparative experiments was performed by a single trained operator using the same calibrated pulse generator system to minimize inter-operator and inter-instrument variability.

Replication strategy and data reporting: All key endpoint experiments (live–dead cell staining, flow cytometry, Western blot, ELISA) were performed with a minimum of three independent biological replicates. A biological replicate is defined as cells cultured, treated, and processed in completely separate sessions (different days, from separate culture passages). Quantitative data from these replicates are presented in all figures as the mean to transparently communicate experimental variability. The statistical methods described below are applied to these biological replicate data to assess significance.

### 2.6. Study Endpoints

The primary endpoints of this study were the ablation efficacy (dose-dependent reduction in cell viability) of PEF on plaque-forming cells and the change in the proportion of Annexin V-positive cells. Secondary endpoints included alterations in cell surface ultrastructure, expression changes in apoptosis-associated proteins, and secretion profiles of selected inflammatory cytokines post-PEF treatment.

### 2.7. Statistical Methods

GraphPad Prism 9.5.1 software was used for all statistical analyses. Quantitative data are presented as the mean from three independent biological replicates (*n* = 3). A biological replicate is defined as cells cultured, treated, and analyzed in separate, independent experimental sessions. Differences between two groups of data were analyzed by the Mann–Whitney U test. The Kruskal–Wallis test was used for multiple comparisons. Where the Kruskal–Wallis test was significant, Dunn’s post hoc test with adjustment for multiple comparisons was applied for pairwise comparisons. A two-tailed *p* value of less than 0.05 was considered statistically significant. Exact *p*-values are reported where feasible.

## 3. Results

### 3.1. Foam Cell Model Construction and Characterization

**Figure 2:**
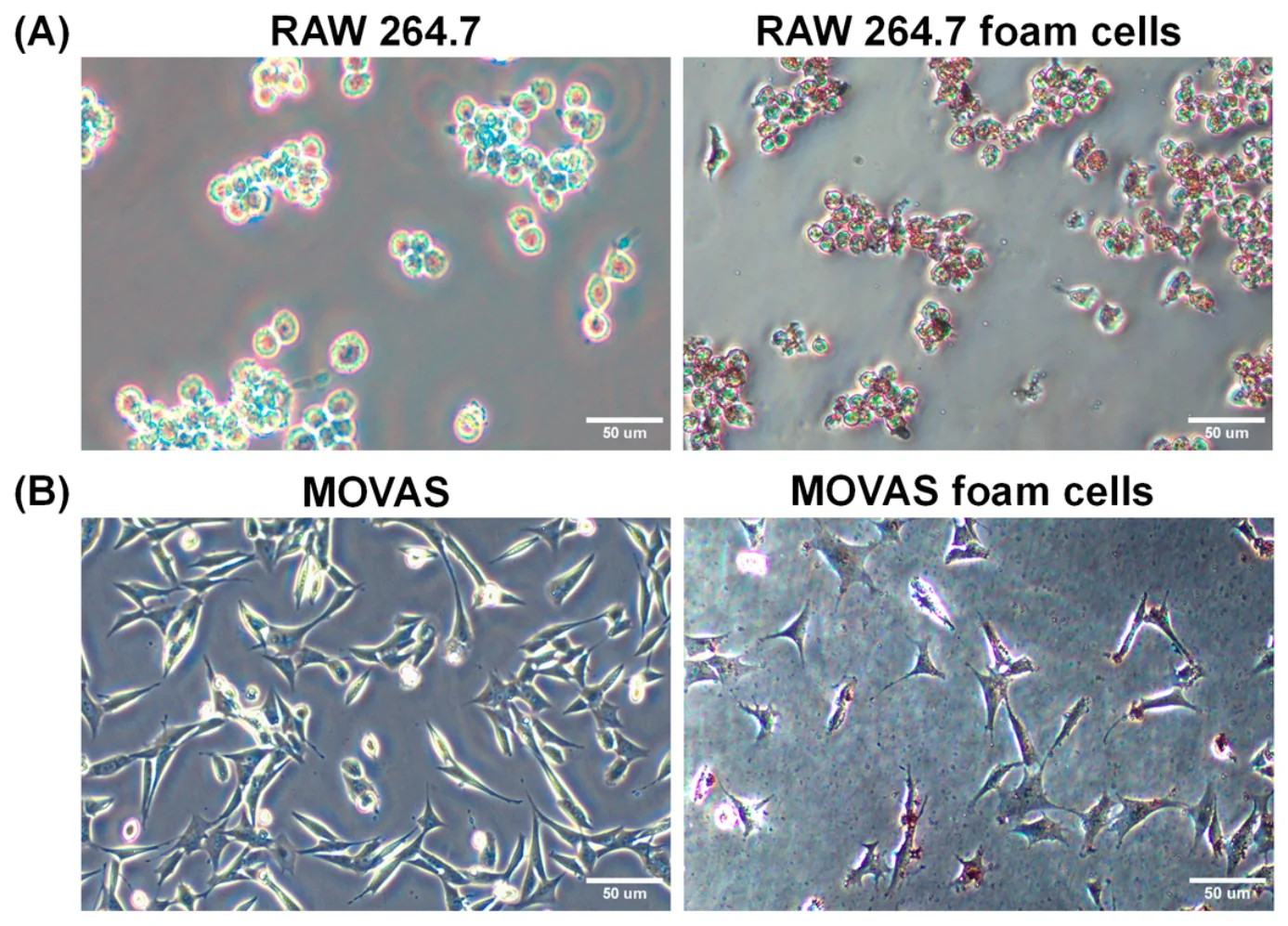
Induction of foam cells from RAW 264.7 (**A**) and MOVAS (**B**), as visualized by Oil Red O staining (200×). The presence of numerous intracellular orange-red lipid droplets confirms the uptake of ox-LDL and the characteristic “foamy” morphology of foam cells. Scale bar is 50 μm. ox-LDL: oxidized low-density lipoprotein.

RAW 264.7 and MOVAS cells were stained with Oil red O 24 h after ox-LDL induction. Normal RAW 264.7 cells are round and uniform in size, with homogeneous and transparent cytoplasm and well-defined margins. After 24 h of induction by ox-LDL, their cell morphology tended to be irregular, and large numbers of orange-red lipid droplets appeared in the cytoplasm (positive Oil red O staining). MOVAS SMCs are typically long and spindle shaped, with well-defined contours and uniformly clear cytoplasm. After 24 h of induction by ox-LDL, their cell morphology was altered, and large numbers of orange-red lipid droplets appeared in the cytoplasm. The dense accumulation of lipid droplets gave RAW 264.7 and MOVAS cells a typical “foamy” appearance (Figure 2).

### 3.2. Effect of PEF Ablation on Cell Viability and Surface Ultrastructure

**Figure 3:**
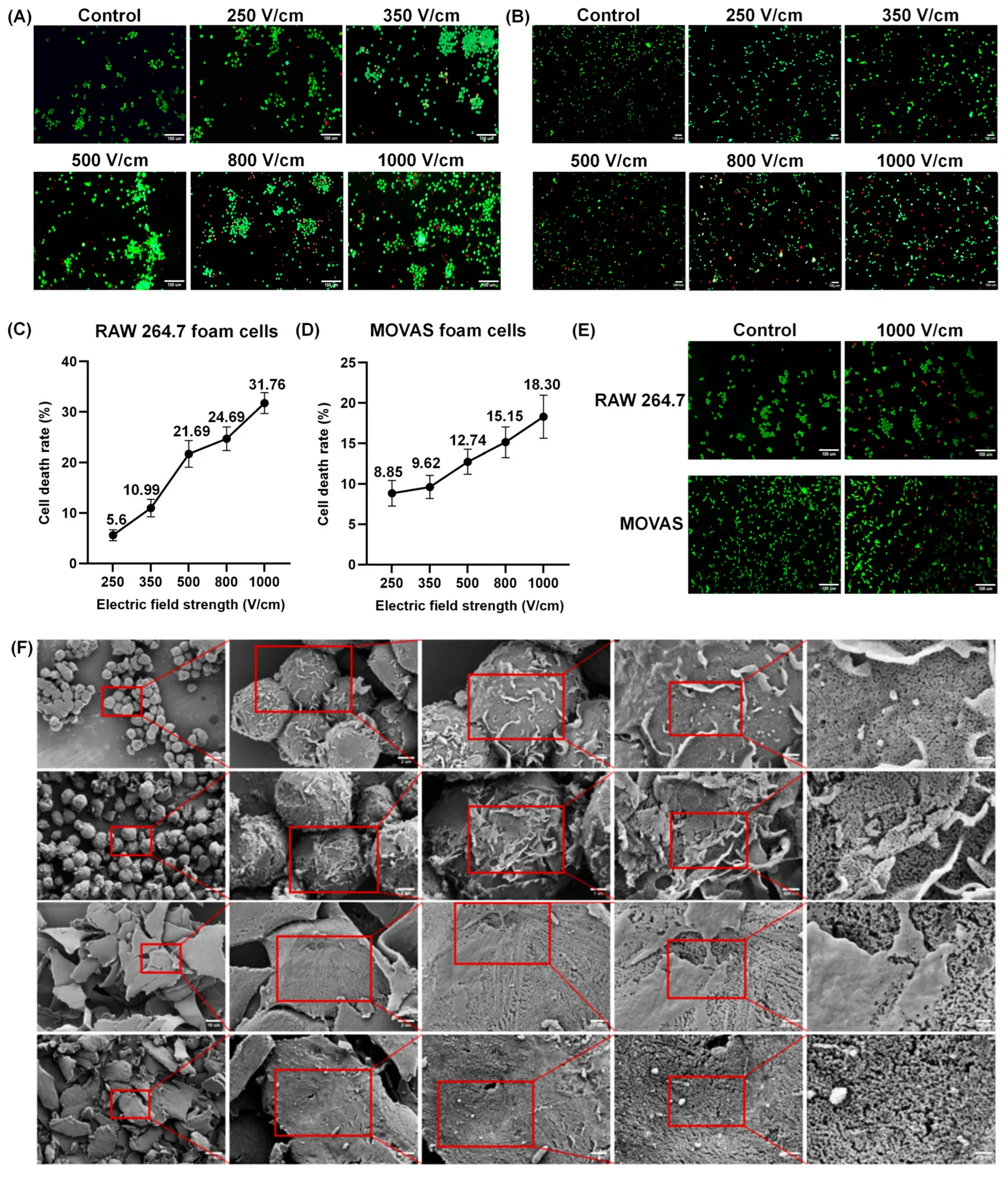
Integrated figure showing the effects of PEF on foam cells and parental cells. (**A**) Representative live–dead cell staining (100×) of RAW 264.7 foam cells 24 h after PEF treatment at indicated electric field strengths. Viable cells (green, Calcein-AM) and dead cells (red, PI) are shown. Scale bar = 100 μm. (**B**) Representative live–dead cell staining (40×) of MOVAS foam cells 24 h after PEF treatment. Scale bar = 100 μm. (**C**,**D**) Dose-dependent effect of PEF on the viability of RAW 264.7 foam cells (**C**) and MOVAS foam cells (**D**), the sample size (*n* = 3 per group). Cell death rates gradually increased with rising electric field strength in the monolayer adherent cell ablation system. (**E**) Representative live–dead staining images (100×) of RAW 264.7 cells and MOVAS cells 24 h after treatment with 1000 V/cm PEF. Scattered bright red fluorescence indicates dead cells. Scale bar = 100 μm. (**F**) Ultrastructural changes in cell membranes observed by scanning electron microscopy (SEM). From top to bottom: untreated RAW 264.7 cells, RAW 264.7 cells 24 h after 1000 V/cm PEF treatment, untreated MOVAS cells, and MOVAS cells 24 h after 1000 V/cm PEF treatment. From left to right, observations at 1.00 K×, 5.00 K×, 10.00 K×, 20.00 K×, and 50.00 K× reveal pronounced membrane ultrastructural changes, including disruption of the surface meshwork and pore-like features. Red boxes indicate areas of local magnification. PEF: pulsed electric field; SEM: scanning electron microscopy.

Under a fluorescence microscope, the live–dead cell staining method can distinguish living and dead cells by staining with Calcein AM/PI. In the live–dead staining of the RAW 264.7 foam cells, the green fluorescently labeled surviving cells were round or round-like with clear boundaries, whereas the red fluorescently labeled dead cells were randomly scattered as dots and were partially attached to the surface of the surviving cells or free on the black background (Figure 3A). In live–dead staining of MOVAS foam cells, surviving cells (green) were irregularly polygonal or spindle shaped, while dead cells (red) were scattered as punctate structures (Figure 3B). As the intensity of the PEF treatment increased, the number of dead cells gradually increased in both groups of foam cells (Figure 3C,D).

We used live–dead cell staining to determine the survival rate 24 h after RAW 264.7 cells and MOVAS cells were ablated by 1000 V/cm PEF. Scattered bright red fluorescence appeared in the images of both RAW 264.7 cells and MOVAS cells after PEF treatment compared with that in the control group (Figure 3E).

SEM analysis revealed that PEF treatment (1000 V/cm) induced pronounced membrane ultrastructural changes in both RAW 264.7 and MOVAS cells 24 h post-treatment. Compared to control cells, which displayed relatively smooth surfaces with uniform porous meshworks, PEF-treated cells of both types exhibited marked morphological disruption (Figure 3F). The overall cell architecture became irregular and rough, with apparent reduction or collapse of membrane ruffles and pseudopods. Most notably, the fine, regular porous network on the cell surface was severely disorganized, accompanied by distorted lamellar protrusions, widened fissures, and haphazardly distributed pore-like features. The apparent pore diameters in PEF-treated cells appeared larger and more irregular (approximately 20–100 nm vs. 10–50 nm in controls). These morphological changes indicate that PEF treatment altered plasma membrane ultrastructure under the present conditions.

### 3.3. Assessment of Annexin V-Positive Cell Proportions by Flow Cytometry

**Figure 4:**
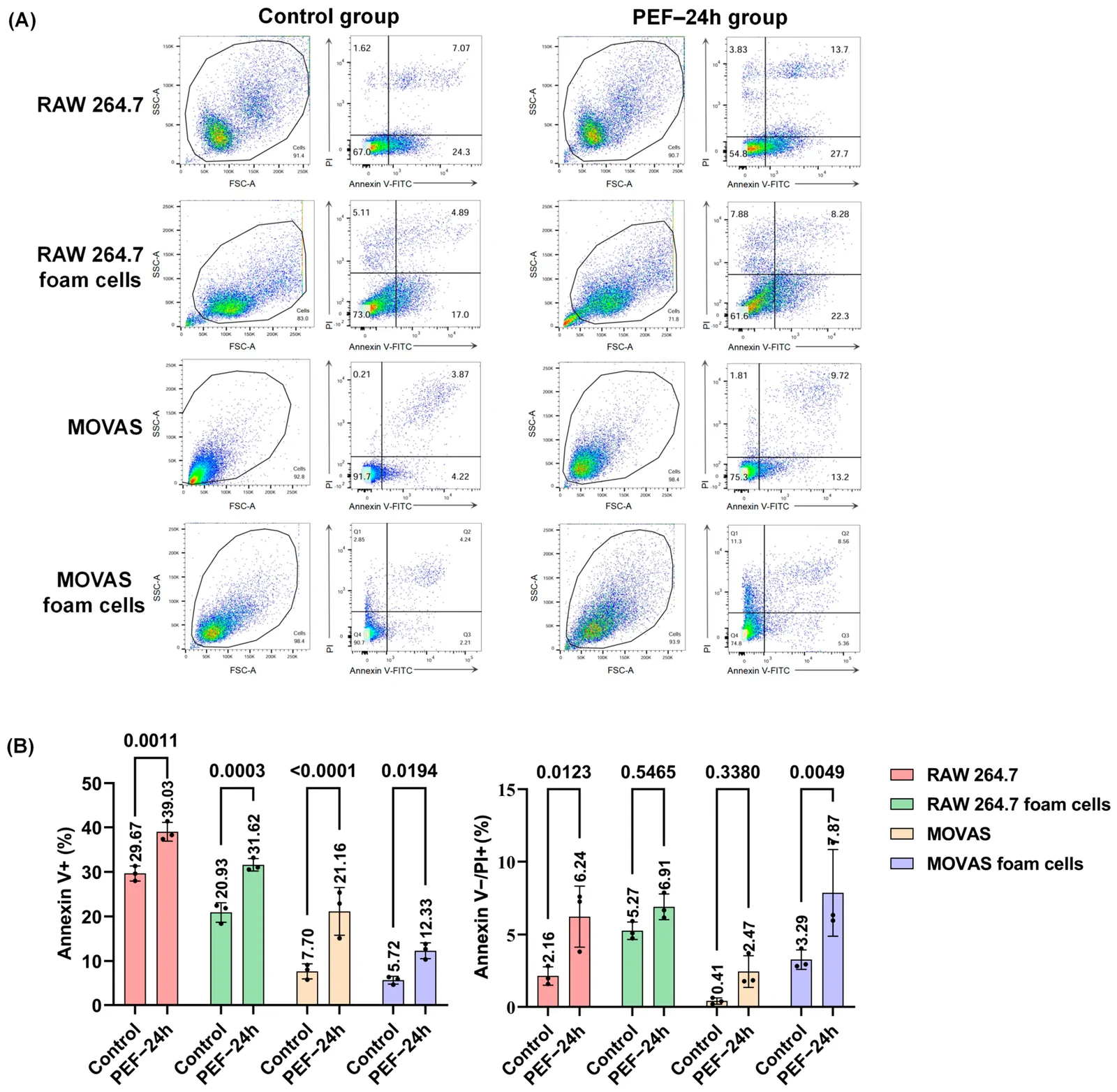
Analysis of cell death mode by Annexin V-FITC/PI flow cytometry. (**A**) Representative flow cytometry dot plots of Annexin V-FITC and PI staining for the indicated cell types 24 h after treatment with or without 1000 V/cm PEF. The lower left quadrant (Annexin V−/PI−) shows viable cells; the lower right quadrant (Annexin V+/PI−) shows early apoptotic cells; the upper right quadrant (Annexin V+/PI+) shows late apoptotic and secondary necrotic cells; the upper left quadrant (Annexin V−/PI+) shows necrotic cells. (**B**) Quantitative summary of the percentages of Annexin V+ and Annexin V−/PI+ cells from the flow cytometry analysis. Data are presented as the mean from three independent biological replicates. PEF: pulsed electric field; PI: propidium iodide.

The proportion of Annexin V-positive cells was quantified using Annexin V-FITC/PI double staining (Figure 4). After PEF treatment (1000 V/cm) at 24 h, the total proportion of Annexin V-positive cells (including Annexin V+/PI− and Annexin V+/PI+ populations) increased in all tested cell types. In RAW 264.7 cells, the Annexin V-positive proportion increased from 29.67% to 39.03%; in RAW 264.7 foam cells, from 20.93% to 31.62%; in MOVAS cells, from 7.70% to 21.16%; and in MOVAS foam cells, from 5.72% to 12.33%. The Annexin V−/PI+ population also showed numerical increases in most cell types (RAW 264.7: 2.16% to 6.24%; RAW 264.7 foam cells: 5.27% to 6.91%; MOVAS: 0.41% to 2.47%; MOVAS foam cells: 3.29% to 7.87%). Because Annexin V+/PI+ cells may include both late apoptotic and secondary necrotic cells, and because singlet discrimination was not performed, these data are reported as descriptive observations of membrane phosphatidylserine externalization. No claim is made that a specific cell death modality (apoptosis vs. necrosis) was definitively identified.

### 3.4. Western Blot Detection of Apoptosis-Related Protein Expression

**Figure 5:**
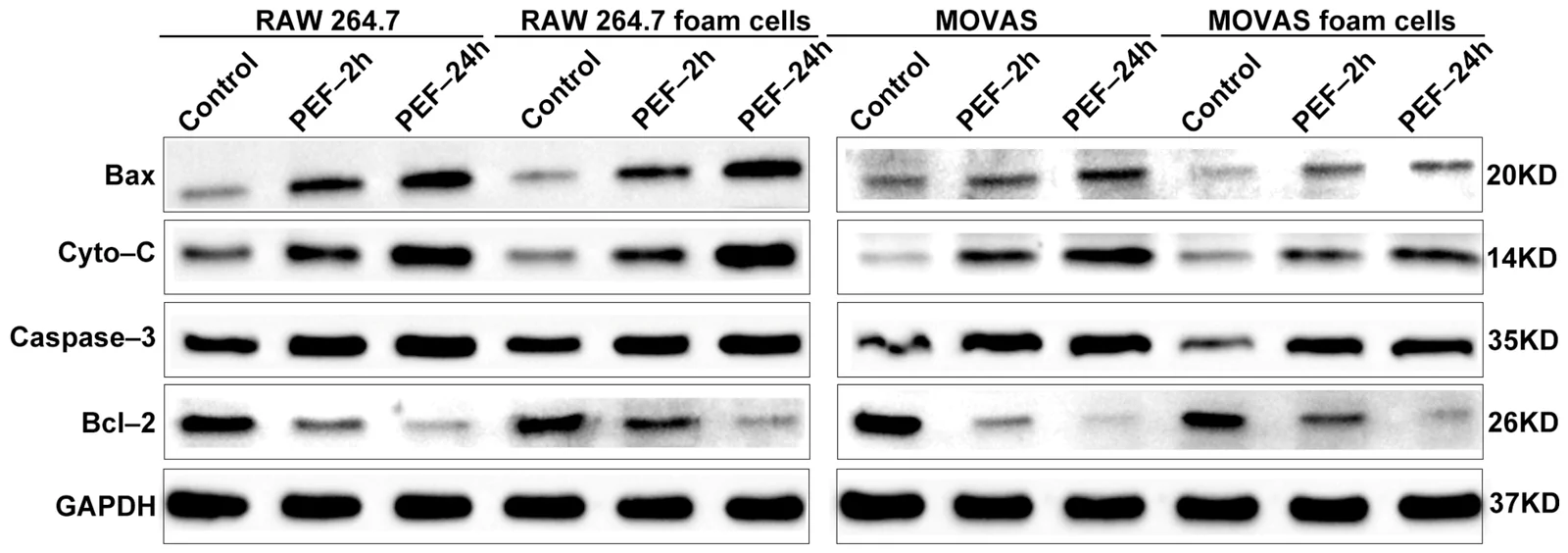
Representative Western blot images showing the expression levels of the apoptosis-related proteins Bax, Cytochrome C, Caspase-3 and Bcl-2 in the indicated cell types, including RAW 264.7, RAW 264.7 foam cells, MOVAS and MOVAS foam cells.

Western blot analysis was used to measure the expression of apoptosis-associated proteins (Bax, Bcl-2, Caspase-3, Cytochrome C) at 2 h and 24 h after PEF treatment (1000 V/cm). Expression changes varied by cell type and time point. In most cell types, Bax, Caspase-3, and Cytochrome C showed increasing trends at 2 h and remained elevated or further increased at 24 h, while Bcl-2 showed a decreasing trend. The magnitude and statistical significance of these changes varied by cell type and time point (Figure 5 and Supplementary Figure S1). Relative protein contents were calculated from grayscale values standardized to internal reference proteins. Because total protein levels were measured, these data are reported as expression changes in apoptosis-associated proteins and do not establish activation of a specific apoptotic pathway.

### 3.5. Effect of PEF Ablation on the Expression of Inflammatory Factors

**Figure 6:**
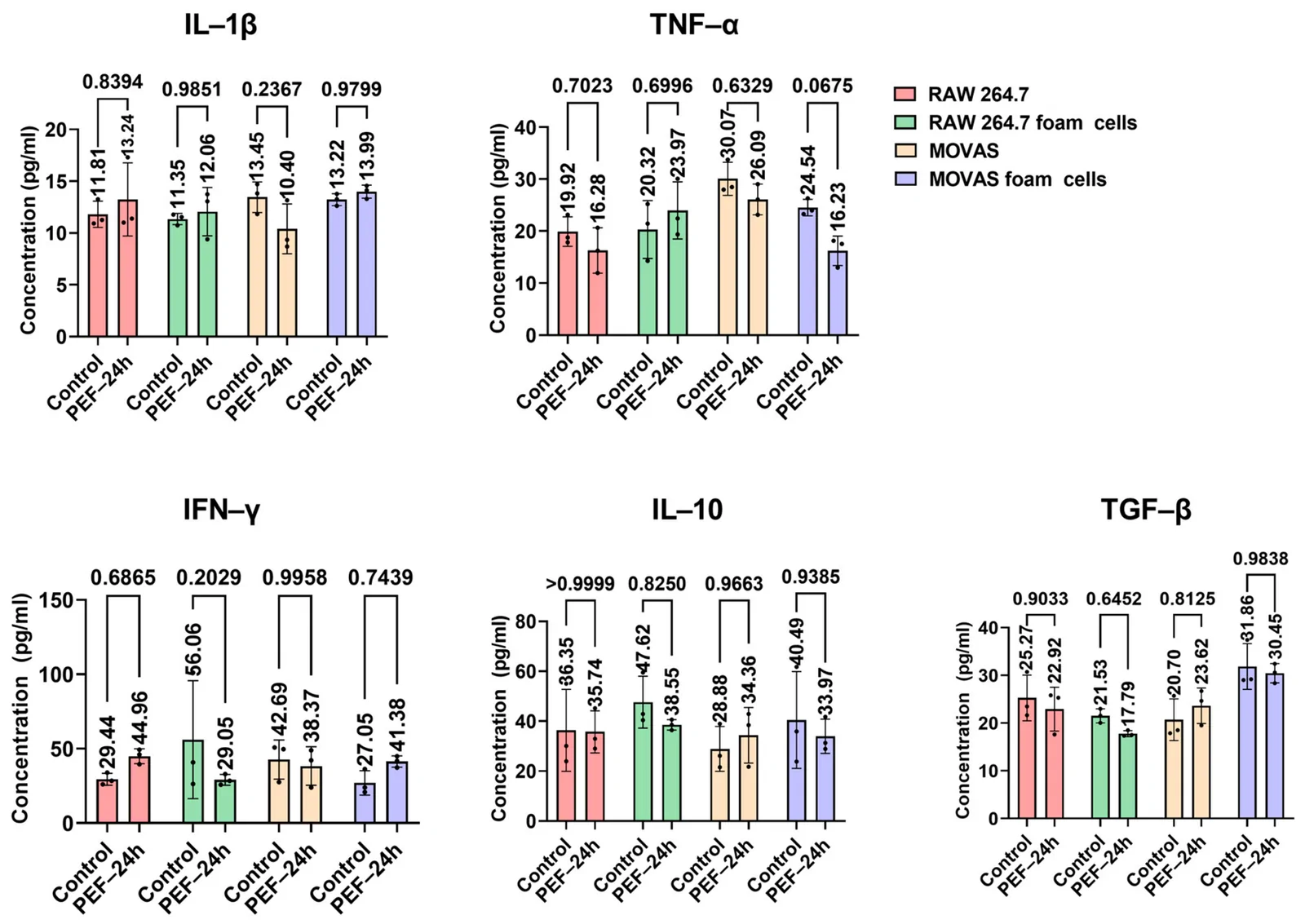
Secretion profile of inflammatory cytokines 24 h after PEF treatment (1000 V/cm). Concentrations of the pro-inflammatory cytokines (IL-1β, TNF-α, IFN-γ) and anti-inflammatory cytokines (IL-10, TGF-β) in culture supernatants were measured by ELISA 24 h after treatment with or without 1000 V/cm PEF. Pink represents RAW 264.7; green represents RAW 264.7 foam cells; yellow represents MOVAS; purple represents MOVAS foam cells. Data are presented as the mean concentration (pg/mL) from three independent biological replicates. PEF: pulsed electric field; ELISA: enzyme-linked immunosorbent assay.

PEF may influence the inflammatory response of cells by regulating the expression of inflammatory factors during the ablation of foam cells. We evaluated a variety of cytokines known to promote or inhibit inflammation, including IL-1β, TNF-α, IFN-γ, IL-10, and TGF-β, to assess overall inflammatory status. Among these cytokines, IL-1β, TNF-α and IFN-γ are pro-inflammatory cytokines, while IL-10 and TGF-β are anti-inflammatory molecules. We used ELISA to assess cell supernatants collected 24 h after PEF treatment (1000 V/cm) to detect the expression levels of the above cytokines. Each group included three independent biological replicates. The results of the assay revealed no statistically significant differences in the expression of the selected pro-inflammatory or anti-inflammatory factors between the control and PEF-treated groups in any of the four cell types (Figure 6).

## 4. Discussion

Atherosclerosis is a multifaceted chronic inflammatory disease, the progression and clinical complications of which are driven by the dysfunction and dysregulated death of key plaque-resident cells, notably macrophages, SMCs, and their lipid-laden derivatives, foam cells [9,10,11]. While conventional treatments focus on risk factor management and mechanical revascularization, novel strategies aimed at directly and safely reducing the plaque cellular burden are of great interest. PEF ablation, an emerging non-thermal modality, has shown promise in oncology and cardiology by inducing targeted cell death via electroporation [6,7]. However, its application to atherosclerosis presents a distinct paradigm: the goal would not be mass ablation, but potentially the induction of a controlled form of cell death. In this preliminary in vitro investigation, PEF at the tested parameters (up to 1000 V/cm) induced dose-dependent reductions in viability in major atherosclerotic plaque-forming cells (macrophages, SMCs, and their foam-cell derivatives). PEF treatment was associated with pronounced membrane ultrastructural changes, increased Annexin V-positive cell proportions, and variable changes in apoptosis-associated proteins. In addition, no statistically significant differences were detected in the selected pro-inflammatory or anti-inflammatory cytokines at 24 h under the tested conditions. These findings provide preliminary in vitro evidence of PEF-induced ablation effects on plaque-associated cells.

In an atherosclerotic rabbit model of our prior in vivo work, the application of PEF (2000 V/cm, 5 µs per phase) to carotid artery plaques induced significant biological effects [12]. Histological assessment revealed marked vascular remodeling, characterized by extensive apoptosis within the plaque’s inner layer and a distinct axial realignment of SMCs and elastic fibers. In contrast, treatment at the lower 1000 V/cm intensity (with or without rapamycin) resulted in subtler changes. These findings illustrate the dose-dependent nature of PEF effects in a complex tissue environment. This highlights a key translational principle: effective PEF ablation in vivo typically requires higher field strengths than those determined in isolated cell studies. The in vivo milieu—with its tissue architecture, hemodynamic factors, and extracellular matrix—can attenuate the effective field at the cellular level, necessitating higher applied intensities for consistent outcomes. This principle is observed in clinical cardiac ablation, where successful pulsed-field ablation for atrial fibrillation employs fields of 1000–2000 V/cm clinically, despite a lower established in vitro cardiomyocyte threshold of 300–550 V/cm.

The selection of 1000 V/cm as the focal parameter for mechanistic exploration was based on the greatest reduction in viability among the tested fields observed by live–dead staining, which showed a progressive, dose-dependent increase in cell death. This does not represent an optimized setting with respect to the apoptosis-to-necrosis balance, which was not fully characterized across the dose range. This parameter represents a balance between achieving effective ablation and, as our data suggest, favoring an Annexin V-positive over an Annexin V-negative outcome under these in vitro conditions. The use of asymmetric biphasic pulses (positive pulse width: 7 µs, negative: 3 µs) with a 5 µs interphase delay was designed based on previous work to mitigate excessive muscle stimulation and cellular electrolysis, aligning with strategies to improve the safety profile of PEF applications [13,14].

The mechanism by which PEF ablation eliminates cells is fundamentally rooted in the interaction of the electric field with the cell membrane, initiating a cascade of biological responses that culminate in cell death. In this study, SEM observations confirmed the initiation of this process via electroporation, revealing characteristic ultrastructural membrane alterations-including disruption of the surface meshwork and pore-like features-consistent with established literature [15,16]. However, the subsequent cellular fate under our specific parameters (1000 V/cm) was not immediate lysis. Flow cytometry showed increased Annexin V-positive cell proportions, and Western blot revealed variable changes in apoptosis-associated proteins, including increased Bax, Caspase-3 and Cytochrome C, and decreased Bcl-2 in most cell types. These observations are consistent with-but do not conclusively establish-involvement of apoptotic pathways. Definitive confirmation would require detection of cleaved/activated Caspase-3 and cytosolic Cytochrome C release, which were not assessed in this study. Existing research clearly demonstrates that the fate of cells following electroporation is not a uniform outcome but rather a dynamic process involving both immediate and delayed death, highly dependent on multiple factors such as pulse parameters, cell and tissue types, and the microenvironment [17,18,19]. This range extends from severe irreversible electroporation (IRE) causing rapid necrosis to various sublethal biological stresses (e.g., ATP depletion, ROS burst, calcium dyshomeostasis) that can activate delayed programmed death pathways such as apoptosis, necroptosis and pyroptosis [20,21,22,23]. Gene expression kinetics further support this protracted complexity, showing transitions from early damage response to apoptosis and potentially other death-related genes over time [24]. Therefore, the increased Annexin V positivity observed in this study represents a phenotypic observation of membrane change, and the underlying death modality requires further investigation.

This mode of action holds particular relevance for atherosclerosis therapy. Apoptosis, when followed by efficient efferocytosis, is generally considered a less inflammatory and more orderly process for cell clearance, standing in stark contrast to the highly pro-inflammatory necrosis that contributes to necrotic core expansion and plaque instability [25]. Therefore, the ability of PEF to increase Annexin V-positive cell proportions without a statistically significant increase in selected inflammatory cytokines at 24 h, as suggested by our in vitro data, could represent a potential approach to reducing plaque cellularity. Nevertheless, the therapeutic rationale for ablating plaque cells is not without potential risks. Apoptotic cells that are not efficiently removed by efferocytosis can undergo secondary necrosis and may expand the necrotic core. In addition, extensive ablation of SMCs could weaken the fibrous cap and undermine plaque stability. Therefore, the net effect of PEF on plaque stability cannot be predicted from the present in vitro data and requires careful in vivo evaluation. Future research utilizing models that more closely mimic physiological conditions is crucial to dissect the temporal dynamics, interactions, and net effects of the various death signals activated post-PEF treatment, which is essential for accurately defining its therapeutic window and risks.

Within the limits of this assay, no statistically significant differences were detected in the secretion of the measured pro-inflammatory (IL-1β, TNF-α, IFN-γ) or anti-inflammatory (IL-10, TGF-β) cytokines at 24 h post-treatment. Given the pivotal role of cytokines like IL-1β and TNF-α in driving plaque inflammation and vulnerability [26], the absence of their upregulation in this in vitro setting is a preliminary indicator. However, these results should be interpreted conservatively: cytokine concentrations were not normalized to surviving cell number, only a single 24 h time point was assessed, and the lack of statistically significant differences does not exclude the possibility of inflammatory changes at other time points or in unmeasured mediators. Future research should focus on multi-time point kinetics as a key future direction.

Although the in vitro system used in this study characterized PEF-induced ablation phenomena in plaque-associated cells, it cannot directly simulate its effects on other components of the vessel wall, including the endothelial layer, collagen fibers and elastin. However, pivotal in vivo studies provide important evidence for the selective action of PEF on vascular structure. The study by Maor et al. in a rat carotid artery model demonstrated that even with nonthermal IRE ablation using electric fields as high as 3500 V/cm, while efficiently eliminating vascular SMCs, the extracellular matrix structure remained undamaged, and the endothelial layer completely regenerated within one week [27]. This study directly proves that, with appropriate parameters, the energy of electroporation can be selectively directed at the cell membrane, thereby ablating cells while intactly preserving the extracellular matrix (ECM) structure that serves as the vascular “scaffold” and allowing for rapid endothelial repair. This finding is consistent with and supportive of our team’s preliminary observations that no acute structural disruption of the fibrous cap was observed post-PEF treatment in atherosclerotic animal models [12]. However, direct evidence in atherosclerotic plaque models is currently lacking. Together, these observations raise the possibility that, with appropriate parameters, PEF may preferentially eliminate pathological plaque cells while preserving ECM structures. This remains a hypothesis requiring direct validation in relevant plaque models. If this hypothesis is confirmed in more complex models, PEF could be explored as a potential local treatment strategy, pending rigorous in vivo validation.

## 5. Study Limitations

This research is still a preliminary exploratory in vitro investigation. Its main purpose is to identify certain phenomena or trends. The conclusions of this study must be interpreted within the context of its inherent limitations, which also chart a clear course for future research.

First and foremost, this is an in vitro investigation using murine cell lines. While this model is essential for establishing proof-of-concept and delineating basic mechanisms, it cannot replicate the complex three-dimensional architecture, hemodynamic shear stress, or multicellular crosstalk of an in vivo human atherosclerotic plaque. Moreover, the immortalized nature of these lines may render them more susceptible to spontaneous cell death than primary cells, which warrants caution in extrapolating absolute cell-death rates.

Second, the inflammatory assessment is limited to a single 24 h time point, a defined set of cytokines, and culture-supernatant ELISA without normalization to viable cell number or total protein content. Inflammatory responses are dynamic. Transient early changes or later shifts in the resolution phase could be missed. Therefore, our conclusion regarding the inflammatory response is specific to the conditions tested and should be viewed as a foundational observation rather than a comprehensive safety profile.

Third, this study did not include vascular endothelial cells. Because each cell type was treated separately and no co-culture or three-dimensional model was used, the present data cannot address the selectivity or spatial targeting of PEF ablation, nor the response of healthy endothelium. Dedicated studies are therefore required to assess endothelial sensitivity and to define a truly selective therapeutic window.

Fourth, this study primarily characterizes the ablation phenomena at a single effective parameter (1000 V/cm). A comprehensive dose–response analysis of cell death modalities across a range of field strengths was not performed, and the 1000 V/cm condition should not be interpreted as an “optimized” parameter.

Fifth, Annexin V/PI staining at 24 h cannot distinguish late apoptosis from secondary necrosis. Annexin V+/PI+ cells may include both populations. Therefore, the proportions of Annexin V-positive cells are reported descriptively without asserting a specific mode of cell death. Additional assays such as lactate dehydrogenase (LDH) release, cleaved/activated Caspase-3 detection, or earlier time point analysis would be needed to definitively dissect the death modality. In addition, conventional singlet discrimination was not performed in flow cytometry because pulse geometry parameters were unavailable.

Sixth, Western blot analyses measured total Bax, Caspase-3, and Cytochrome C protein levels, rather than cleaved/activated Caspase-3, cleaved Poly ADP-ribose polymerases (PARP), or cytosolic Cytochrome C. The observed changes are therefore consistent with—but do not conclusively establish—activation of the mitochondrial apoptotic pathway.

Finally, several methodological parameters were not directly measured: pulse impedance, delivered energy, and temperature changes during treatment were not recorded, and the electric field strength was calculated from applied voltage and electrode distance rather than measured directly. These factors should be addressed in future studies.

## 6. Conclusions

In summary, this preliminary in vitro investigation demonstrates that PEF, at the tested parameters (up to 1000 V/cm, asymmetric biphasic pulses), induces dose-dependent ablation effects on key atherosclerotic plaque-forming cells—macrophages, SMCs, and their foam-cell derivatives. PEF treatment was associated with pronounced membrane ultrastructural changes, increased Annexin V-positive cell proportions, and variable changes in apoptosis-associated proteins. No statistically significant differences were detected in the selected cytokines at 24 h under the tested conditions. The precise mode of PEF-induced cell death, the underlying molecular mechanisms, and the in vivo relevance of these findings require further investigation in more physiologically relevant models and animal studies.

## Data Availability

The datasets generated during and/or analyzed during the current study are available from the corresponding author upon reasonable request.

## Supplementary Materials

The following supporting information can be downloaded at: https://www.mdpi.com/article/10.3390/jcdd13100515/s1, Figure S1: Densitometric quantification of key apoptosis-related proteins in (A) RAW 264.7 and RAW 264.7 foam cells, and (B) MOVAS and MOVAS foam cells, at 2 h and 24 h after PEF treatment (1000 V/cm). PEF treatment significantly increased the levels of pro-apoptotic proteins (Bax, Cytochrome C, Caspase-3) and decreased the level of the anti-apoptotic protein Bcl-2.

## Abbreviations

The following abbreviations are used in this manuscript:

ASCVDs	atherosclerotic cardiovascular diseases
DMEM	Dulbecco’s modified eagle medium
ECL	enhanced chemiluminescence
ECM	extracellular matrix
ELISA	enzyme-linked immunosorbent assay
FSC-A	forward scatter
HRP	horseradish peroxidase
Hz	Hertz
IRE	irreversible electroporation
ox-LDL	oxidized low-density lipoprotein
PEF	pulsed electric field
PI	propidium iodide
PARP	Poly ADP-ribose polymerases
SE	standard error
SEM	scanning electron microscopy
SMCs	smooth muscle cells
SSC-A	side scatter
